# Effects of maternal advanced lipoxidation end products diet on the glycolipid metabolism and gut microbiota in offspring mice

**DOI:** 10.3389/fnut.2024.1421848

**Published:** 2024-06-19

**Authors:** Wenwen Pang, Bowei Zhang, Junshi Zhang, Tianyi Chen, Qiurong Han, Zhen Yang

**Affiliations:** ^1^Department of Clinical Laboratory, Tianjin Union Medical Center, Nankai University, Tianjin, China; ^2^School of Medicine, Nankai University, Tianjin, China; ^3^Department of Hematology, Oncology Center, Tianjin Union Medical Center, Nankai University, Tianjin, China; ^4^School of Integrative Medicine, Tianjin University of Traditional Chinese Medicine, Tianjin, China

**Keywords:** maternal diet, advanced lipoxidation end products, offspring, glycolipid metabolism, gut microbiota

## Abstract

**Introduction:**

Dietary advanced lipoxidation end products (ALEs), which are abundant in heat-processed foods, could induce lipid metabolism disorders. However, limited studies have examined the relationship between maternal ALEs diet and offspring health.

**Methods:**

To investigate the transgenerational effects of ALEs, a cross-generation mouse model was developed. The C57BL/6J mice were fed with dietary ALEs during preconception, pregnancy and lactation. Then, the changes of glycolipid metabolism and gut microbiota of the offspring mice were analyzed.

**Results:**

Maternal ALEs diet not only affected the metabolic homeostasis of dams, but also induced hepatic glycolipid accumulation, abnormal liver function, and disturbance of metabolism parameters in offspring. Furthermore, maternal ALEs diet significantly upregulated the expression of TLR4, TRIF and TNF-α proteins through the AMPK/mTOR/PPARα signaling pathway, leading to dysfunctional glycolipid metabolism in offspring. In addition, 16S rRNA analysis showed that maternal ALEs diet was capable of altered microbiota composition of offspring, and increased the Firmicutes/Bacteroidetes ratio.

**Discussion:**

This study has for the first time demonstrated the transgenerational effects of maternal ALEs diet on the glycolipid metabolism and gut microbiota in offspring mice, and may help to better understand the adverse effects of dietary ALEs.

## 1 Introduction

The heat-processed foods contain high amounts of advanced lipoxidation end products (ALEs), which are crosslinked products of active carbonyl compounds produced by lipid oxidation and nucleophilic groups in proteins (1). In recent decades, overconsumption of heat-processed foods greatly increases the burden of chronic health conditions. The offspring of dams who fed with different types of heat-processed foods had lower birth weight (2) and increased catch-up growth (3) delayed maturation of physical features and impaired glucose homeostasis (4). Therefore, maternal ALEs diet has profound and lasting effects on the long-term health and disease risks of offspring.

It is known that the maternal nutrition and intrauterine environment play a key role in the development of metabolic disease in offspring (5). Recent studies on the underlying mechanisms of this programming effect have found that maternal diets can affect insulin and glucose metabolism, energy balance, cardiovascular function, and obesity in offspring (6, 7). In addition, there is evidence that changes in epigenetic marks are one mechanism that could account for such long-term effects, as they affect gene expression and thus shape the phenotype of the organism (8). Accordingly, the adverse consequences caused by the dietary ALEs are not limited to themselves, but may also have corresponding effects on the health of their offspring, which deserves attention.

ALEs can be formed exogenously or endogenously (9), and endogenous ALEs are closely related to physiological processes, such as oxidative stress, inflammation and liver injury (10). Exogenous ALEs are known as dietary ALEs, which are produced by reaction during food hot processing, and are considered as potential health risk factors. Our previous research has reported that dietary ALEs which are formed by reactive carbonyl compounds modified proteins have been shown not only resulting in alterations in the protein structure and digestibility, but also to cause abnormal liver function and lipid accumulation in mice (11). Dietary ALE is formed by modifying amino acids (lysine, cysteine, arginine and histidine) with active carbonyl compounds (malondialdehyde, 4-hydroxy-2-nonenal, etc.) in food (12). Since the ALEs-modification alters the structure, it changes the protein function. Binding of ALEs to its specific cell surface receptor-receptor for advanced glycation end product (RAGE) initiates downstream pathways leading to the production of reactive oxygen species and inflammatory cytokines, leading to inflammatory or diabetic responses (13, 14). We hypothesized that consumption of an ALEs-rich diet by dams may lead to changes in the metabolism and oxidation status of their offspring. In addition, the glycolipid metabolism of the offspring is closely related to its gut microbiota. To the best of our knowledge, the transgenerational effects of maternal ALEs diet have not been studied so far.

In this study, we continue to investigate the adverse effects of dietary ALEs. A cross-generation mouse model was developed to assess the effects of maternal exposure to dietary ALEs on metabolic disorders in offspring. The results suggested that exposure to maternal ALEs-rich diet might affect offspring’s body weight, glucose and lipid metabolism, and gut microbiota. This study investigated the transgenerational effects of maternal ALEs diet on metabolism, and highlighted the regulatory role of maternal ALEs diet on gut microbiota of the offspring.

## 2 Methods

### 2.1 Animals and diets

The C57BL/6 J (5-week-old, female) mice were purchased from Beijing Vital River Laboratory Animal Technology Co., Ltd. (Beijing, China) and housed in the Nankai University Laboratory Animal Center under controlled conditions (temperature, 20–24°C; humidity, 50–60%; 12 h light/dark cycle). The animals and protocols for this study were approved by international ethical guidelines and the Institutional Animal Care and Use Committee of Nankai University.

The dietary ALEs given to the mice were prepared as we previously published (11). Malondialdehyde and myofibrillar proteins were selected to produce dietary ALEs by a heat-processing simulation system. After 1 week of environmental adaptation, all female mice were randomly divided into 3 groups (*n* = 8), as follows: the low dose ALEs (LALEs) group (3% casein in the standard AIN-93G diet was replaced with ALEs), the normal diet control (CON) group (standard AIN-93G diet), and high dose ALEs (HALEs) group (30% casein in the standard AIN-93G diet was replaced with ALEs). The female mice were fed for a total of 11 weeks, including preconception, pregnancy and lactation. The mice were allowed access to food and water *ad libitum* throughout the study, and their daily food consumption was estimated by weighing the remaining food. As previously reported in our research, the dose could reflect the intake of different doses of human dietary ALEs.

The female mice were mated with male mice for 4 days (female: male = 2:1), and the pregnancy was confirmed by postcopulatory plugs. The number of offsprings in each litter was 6–10. To avoid nutrition bias between litters, the offsprings sizes were culled to 6 for each dam at birth (15, 16). The body weight of the dams and offsprings was measured every week. After 3-week of weaning, the offsprings were anesthetized (*n* = 8, one offspring per litter), and sacrificed to collect serum, liver, and fecal samples.

### 2.2 Glucose tolerance tests in dams and offspring

To avoid stress during pregnancy and lactation, the dams were subjected to intraperitoneal glucose tolerance tests (IPGTTs) after weaning their offsprings. Besides, at 3 weeks of age, IPGTTs were performed on the offsprings after 10 h of fasting. After glucose administration (2 g/kg body weight), blood glucose (BG) levels in the tail vein were monitored using a Contour TS glucometer (Bayer, Beijing, China) before the injection (0 min) and at 30, 60, and 120 min after the injection. The area under the curve (AUC) of IPGTTs was calculated by the trapezoid formula: AUC = 0.5 × (BG0 + BG30)/2 + 0.5 × (BG30 + BG60)/2 + 1 × (BG60 + BG120)/2.

### 2.3 Serum biochemical analysis

The blood samples were collected and centrifuged at 4000 g for 10 min, and the serum was stored at −80°C. The total cholesterol (TC), triglyceride (TG), low-density lipoprotein cholesterol (LDL-C), and high-density lipoprotein cholesterol (HDL-C) in serum were determined using Nanjing Jiancheng Institute of Bioengineering ELISA kits (Nanjing, China). The level of serum fasting insulin (90,080; Crystal Chem, Downers Grove, United States), leptin (MOB00B, R&D Systems, Minneapolis, United States) were measured by ELISA kits. The insulin sensitivity was assessed using the homeostasis model assessment of insulin resistance (HOMO-IR). The HOMO-IR was calculated as the fasting insulin concentration (μU/mL) × fasting glucose concentration (mmol/L)/22.5.

### 2.4 Histopathological observation

For histological study, the fresh liver tissues were fixed in 4% paraformaldehyde, embedded in paraffin and then cut into 5 μm slices. After deparaffinization and hydration, hematoxylin and eosin (H&E) staining (Servicebio) was performed. For alcian blue periodic acid–Schiff (AB-PAS) staining (Servicebio), frozen liver samples were first processed using a cryostat, then fixed, and stained. The images of the sections were captured using fluorescent inverted microscope.

### 2.5 Western blot analysis

Western blotting was applied to determine the expression levels of proteins, including AMPK, mTOR, PPARα, PKM2, IRS-1, TLR4, TRIF and TNF-α in the liver tissue of offspring mice. Total proteins were prepared from frozen liver tissue by Cellytic M cell lysis. The supernatant was loaded on SDS-PAGE gels. After electrophoresis, it was transferred to PVDF membrane (Millipore). The membrane was then incubated at 4°C with the antibodies (Abcam), and β-actin was used as internal load control. After washing, the membrane was incubated with secondary antibodies at room temperature for 1 h. The images were captured by a ChemiDoc MP imaging system (Bio-Rad, Hercules, CA, United States).

### 2.6 Microbial samplings and DNA isolation

High-throughput sequencing of the 16S rRNA gene was used to analyze the distribution of gut microbiota in the feces of maternal and offspring mice. The FastDNA Soil Spin kit (MP Biomedicals, CA, United States) was used to extract microbial genomic DNA from the feces of weaned offspring according to the manufacturer’s protocol. Primer pairs were used to amplify the V3-V4 region of the bacterial 16S rRNA gene, followed by purification of PCR products using the AxyPrep DNA Gel Extraction kit (Axygen Biosciences, United States). Quantification was performed using Quantus^TM^ fluorometer (Promega, United States). Purified amplicons were sequenced on an Illumina NovaSeq PE250 (Illumina, United States). The original FASTQ files are multiplexed using internal perl scripts, then quality filtered through Fastp, and merged through FLASH. The classification information was annotated using the Silva database based on the RDP classifier.

The alpha diversity including richness (Chao1 and Ace) and evenness (Simpson and Shannon) was calculated with mothur, and QIIME software was used to determine the community composition of each sample at different classification levels and beta diversity. The Wilcoxon rank sum test was used to determine the distinct species (phylum and genus) between the two groups. In addition, the linear discriminant analysis (LDA) effect size (LEfSe) was used to evaluate the influence of the abundance of each species on the differences between groups. Based on Kyoto Encyclopedia of Genes and Genomes (KEGG) database, phylogenetic Investigation of Communities by Reconstruction of Unobserved States (PICRUSt 2) was used to predict metagenome function.

### 2.7 Statistical analysis

Data were analyzed using GraphPad Prism (version 9.0, GraphPad Software, United States). The data were presented as mean ± standard error of the mean (SEM) for normally distributed data. Statistical analyses were performed with the normality test, one-way ANOVA, two-way ANOVA and Tukey *post-hoc* test. Correlation analyses between the relative abundance of bacterial taxa was analyzed by Spearman correlation coefficient test. A *p*-value <0.05 was considered statistically significant.

## 3 Results

### 3.1 The effects of maternal ALEs diet on glucose and lipid metabolism in dams

**Figure 1:**
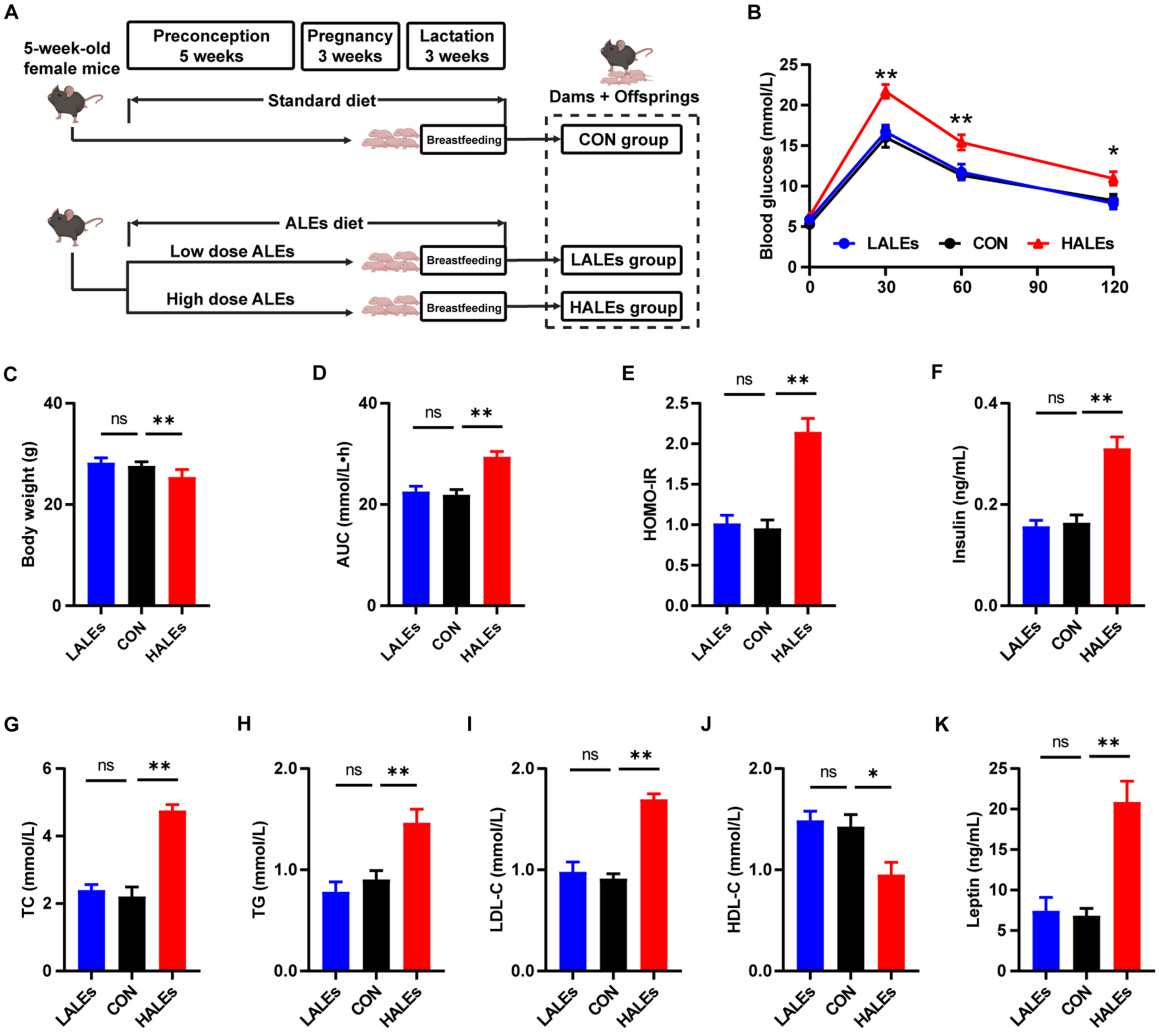
Dietary ALEs impaired glycolipid metabolism in dams. **(A)** The flowchart of this study. **(B)** IPGTTs of dams. **(C)** Body weight of dams. **(D)** AUC of dams. **(E)** HOMO-IR of dams. **(F)** Serum insulin level of dams. **(G)** Serum TC level of dams. **(H)** Serum TG level of dams. **(I)** Serum LDL-C level of dams. **(J)** Serum HDL-C level of dams. **(K)** Serum leptin level of dams. *p < 0.05, **p < 0.01, Data were expressed as the mean ± SEM.

The female mice were fed with the ALEs diet or standard diet during preconception, pregnancy and lactation. The flowchart of this study is presented in Figure 1A. There was no difference in the amount of food intake between the three groups. After 11 weeks of diet intervention, the HALEs diet negatively impacted body weight, and the LALEs diet did not affect the body weight in dams at weaning (Figure 1C).

To investigate whether the ALEs diet impacted glucose and lipid metabolism, we performed the IPGTTs on dams when they weaned their offspring (Figure 1B). The results of IPGTTs showed that HALEs diet led to significantly higher blood glucose levels in dams at 30 min, 60 min and 120 min, as well as a trend toward the larger area under the curve (AUC) values than those of dams in the LALEs and CON group (Figure 1D). Additionally, the HOMO-IR in the HALEs group was significantly higher than the CON group (Figure 1E).

Then we assessed the serum insulin concentration in dams, and found that HALEs diet significantly increased the serum fasting insulin levels, compared with the CON group (Figure 1F). Meanwhile, there were no significant differences in insulin levels between the LALEs and CON groups. The effects of ALEs diet on serum lipid profiles were also evaluated. The serum levels of TC, TG and LDL-C in dams fed by HALEs diet were higher than those fed the standard diet (Figures 1G–I). Besides, the HALEs diet significantly reduced the HDL-C levels (Figure 1J). However, no significant difference was observed in the lipid profiles between the dams in the LALEs group and in the CON group. Furthermore, the HALEs diet also significantly elevated the serum leptin levels (*p* < 0.05) in dams compared with those from the CON group (Figure 1K). These results suggested that ALEs diet could lead to glucose and lipid metabolism disturbance and even resistance in dams.

### 3.2 Effects of maternal ALEs diet on glucose and lipid metabolism in offspring

**Figure 2:**
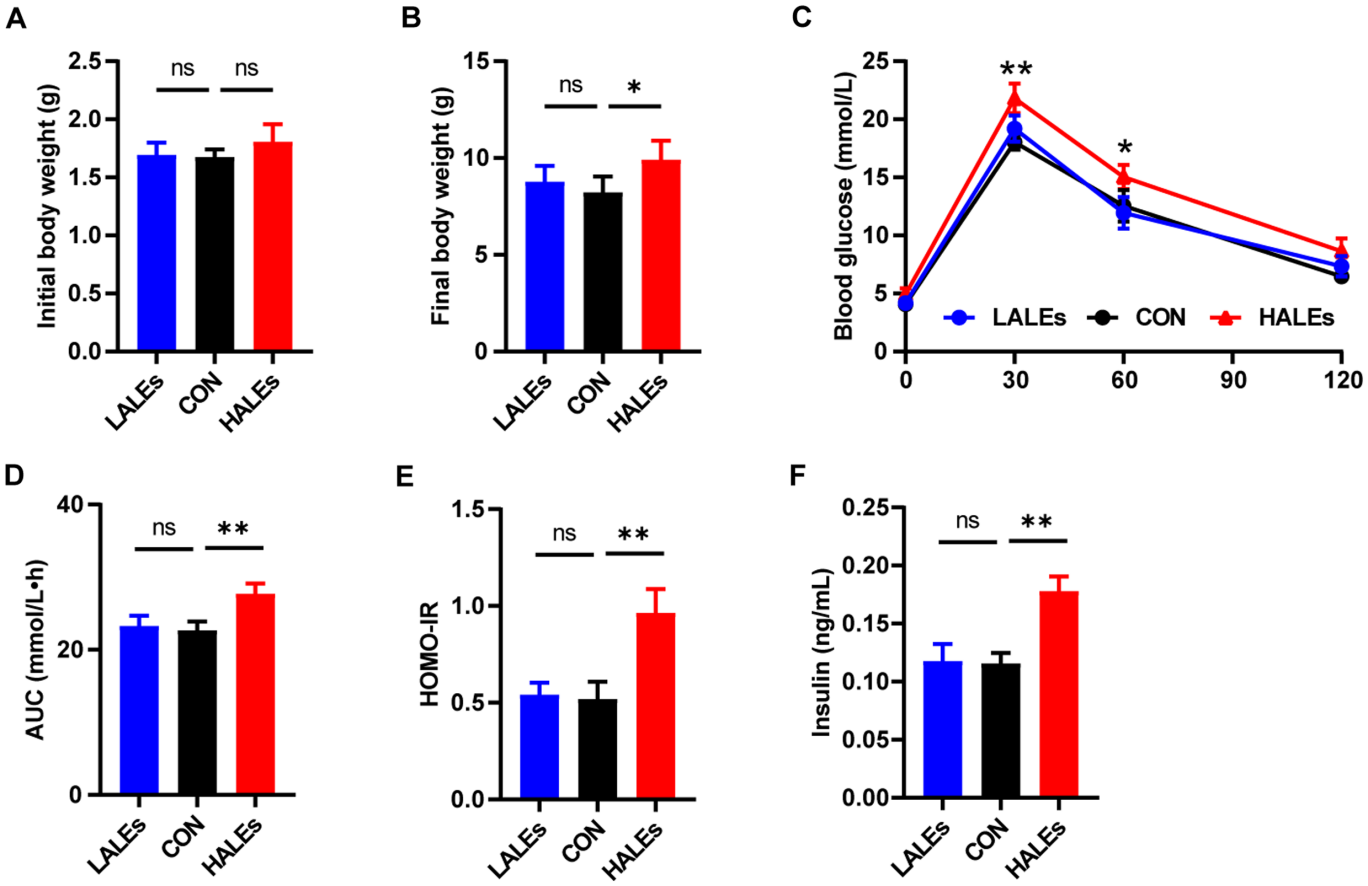
Maternal ALEs diet impaired glucose metabolism in offspring. **(A)** Body weight of offspring at birth. **(B)** Body weight of offspring at weaning. **(C)** IPGTTs of offspring. **(D)** AUC of offspring. **(E)** HOMO-IR of offspring. **(F)** Serum insulin level of offspring. *p < 0.05, **p < 0.01, Data were expressed as the mean ± SEM.

To determine the transgenerational effects of dietary ALEs, we first investigated the phenotype of offsprings. The body weight of offsprings was measured every 3 days from birth to weaning. Our results have shown that no significant differences was observed in the initial body weight (body weight at birth) between the three groups (Figure 2A). Noteworthy, the final body weight (body weight at weaning) of the HALEs group was significantly greater than that of the CON and LALEs groups (Figure 2B). It is observed that the 11-week maternal HALEs diet did affect the body weight of offsprings at weaning.

The IPGTTs was performed on the offspring at weaning. Compared with the CON group, the HALEs group had significantly higher blood glucose levels (Figure 2C). As shown in Figure 2D, the AUC in the HALEs group was significantly larger. There was no difference in the AUC between the LALEs and CON group. In addition, the offspring of the HALEs group had a higher HOMO-IR index compared with the CON group (Figure 2E). To determine whether the HALEs diet reduced insulin sensitivity in the offspring, we assessed the fasting insulin levels. The serum insulin level was significantly higher in the HALEs group (Figure 2F). These results showed that the offspring of the HALEs group exhibited glucose intolerance.

**Figure 3:**
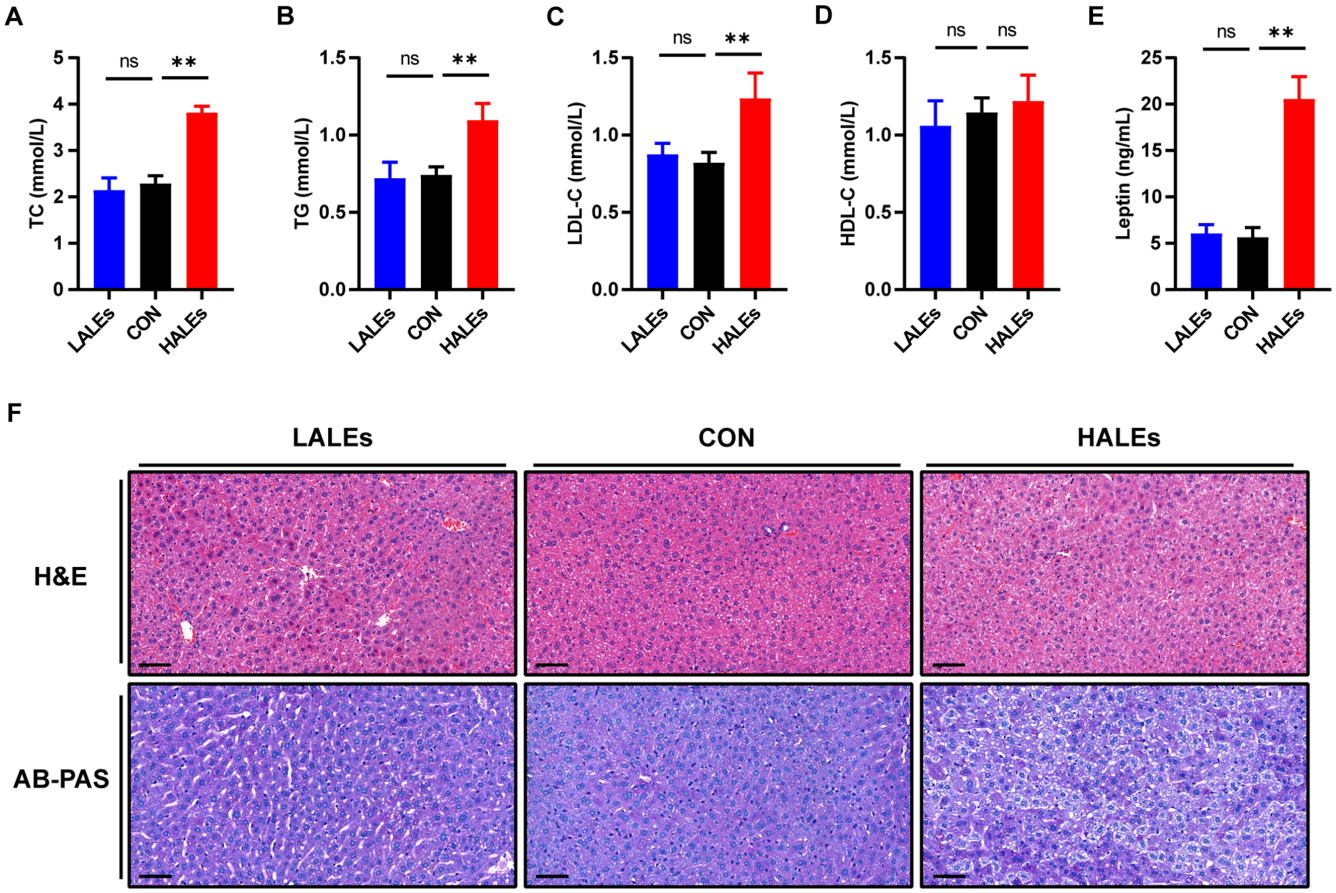
Maternal ALEs diet impaired lipid metabolism in offspring. **(A)** Serum TC level of offspring. **(B)** Serum TG level of offspring. **(C)** Serum LDL-C level of offspring. **(D)** Serum HDL-C level of offspring. **(E)** Serum leptin level of offspring. **(F)** Representative images of H&E-stained and AB-PAS stained liver tissue (200×, bar = 50 μm). *p < 0.05, **p < 0.01, Data were expressed as the mean ± SEM.

In addition, we evaluated the effects of maternal ALEs diet on serum lipid profiles in offspring. The serum levels of TC (Figure 3A) and TG (Figure 3B) were both significantly increased in the offsprings due to the maternal ALEs diet, compared with the CON group. Furthermore, the offspring of the HALEs group displayed higher LDL-C level (Figure 3C). As shown in Figure 3D, the HDL-C level of offspring were not significantly different between the three groups. However, the offspring of the HALEs group had significantly higher serum leptin levels than the offspring of the LALEs and CON groups (Figure 3E), which suggested that maternal ALEs diet induced lipid metabolism disorder in offspring.

### 3.3 Effects of maternal ALEs diet on liver function in offspring

To explore the impact of maternal ALEs diet on liver damage in offspring, histopathology profiles were observed by H&E and AB-PAS staining. As shown in Figure 3F, the offspring liver ultrastructure of LALEs and CON group was normal, and there was no steatosis. The hepatocytes were arranged neatly, and their cords were arranged radially around the central vein. However, the offspring liver of the HALEs group was characterized by deformed and compressed hepatocytes, disorganized cords, and cytoplasmic accumulation of lipid droplets with varied size, number and shape. The AB-PAS staining showed that the glycogen content in the HALEs group was significantly increased compared with the CON group. The results indicated that maternal ALEs diet induced liver damage, liver lipid accumulation, and increased liver glycogen content in offspring.

**Figure 4:**
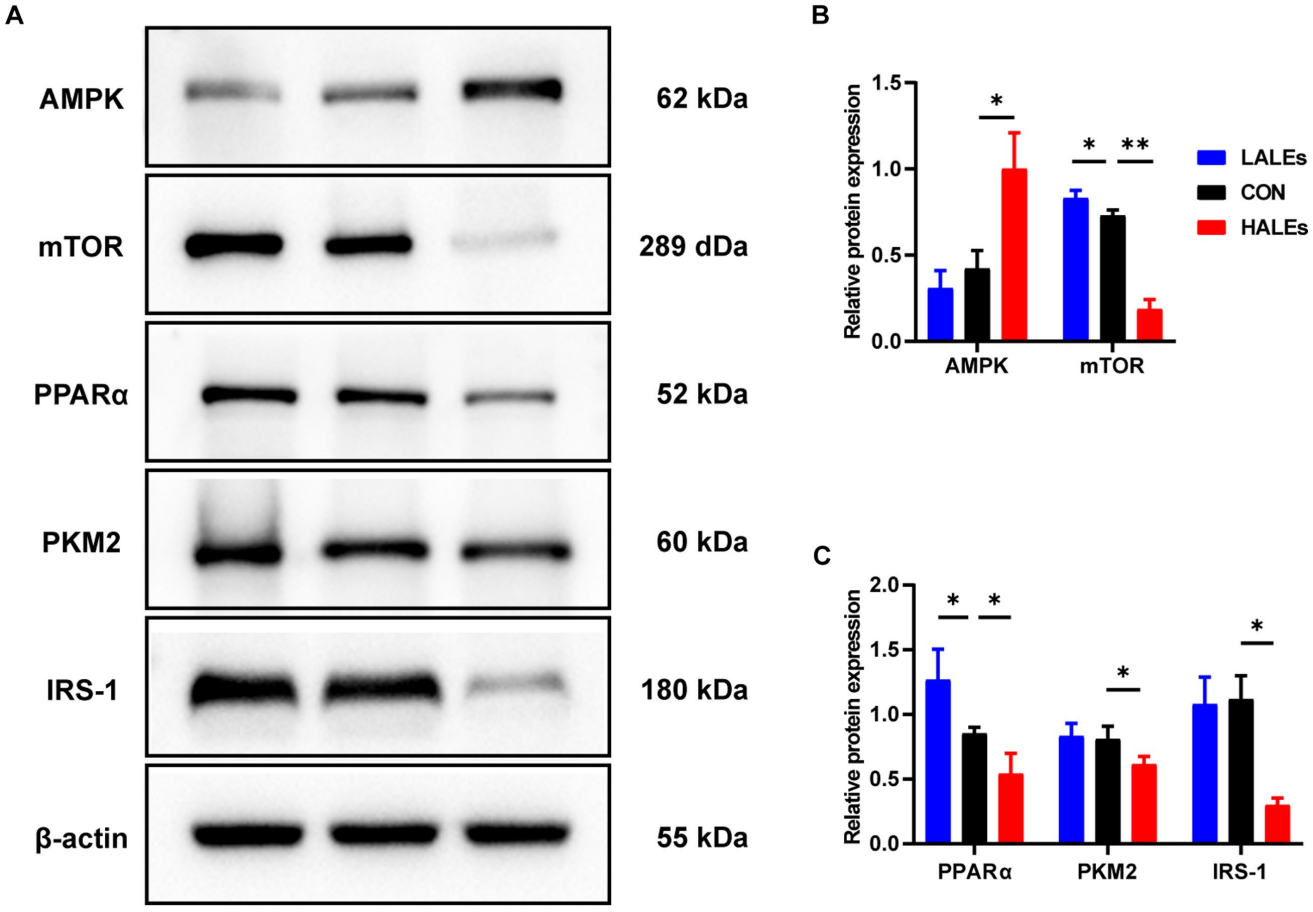
Maternal ALEs diet regulated the AMPK/mTOR/PPARα signaling pathway. **(A)** Protein expression measured by Western blot. **(B)** Protein expression of AMPK and mTOR in the liver. **(C)** Protein expression of PPARα, PKM2 and IRS-1 in the liver. *p < 0.05, **p < 0.01, Data were expressed as the mean ± SEM.

**Figure 5:**
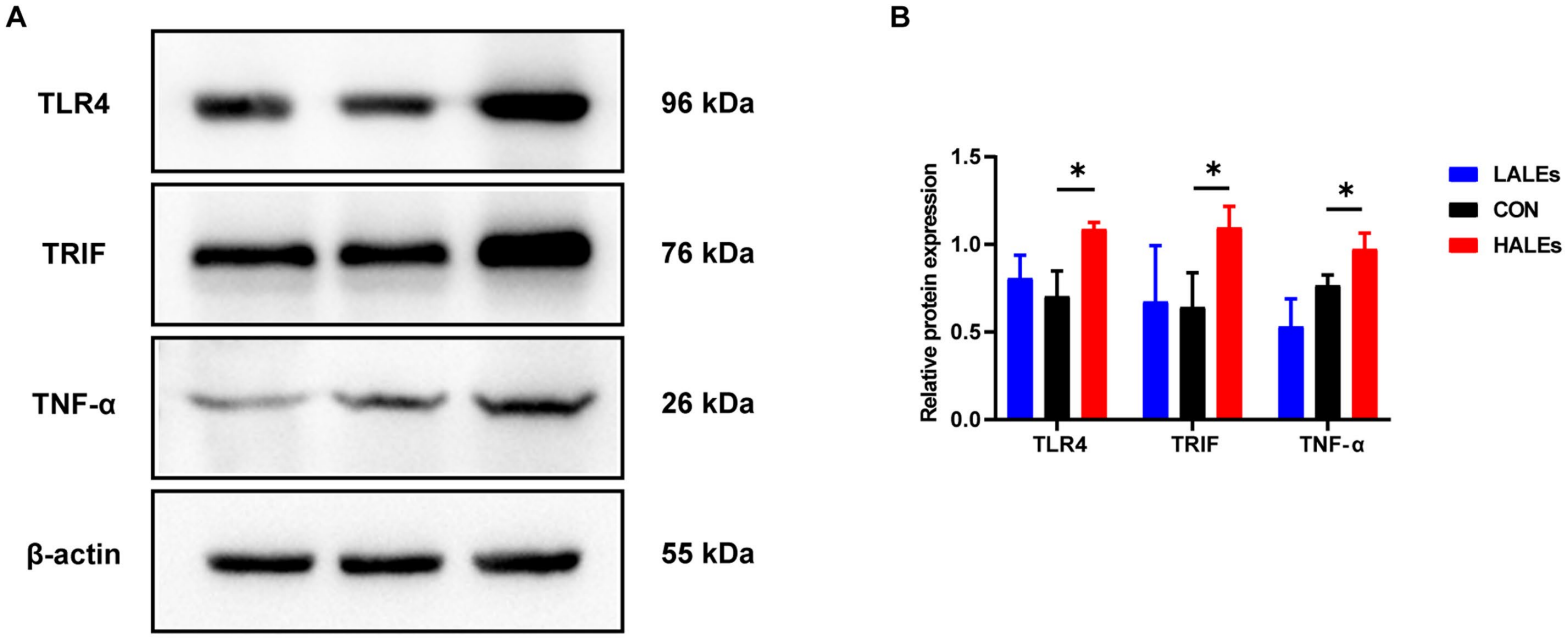
Maternal ALEs diet influenced the expression of TLR4/TRIF/TNF-α proteins. **(A)** Protein expression measured by Western blot. **(B)** Protein expression of TLR4, TRIF and TNF-α in the liver. *p < 0.05, **p < 0.01, Data were expressed as the mean ± SEM.

We further investigated the molecular mechanism of glucose lipid metabolism disorder induced by maternal ALEs diet in offspring. As the liver is the most important organ of metabolism, the expression of proteins involved in glucose and lipid metabolism in the liver was examined by Western blot (Figure 4A). As shown in Figure 4B, the expression of AMPK was significantly increased in the HALEs group compared with the CON group. The expression of mTOR, PPARα, PKM2 and IRS-1 in the HALEs group was significantly lower than those in the CON group (Figures 4B,C). In addition, the maternal HALEs diet significantly increased the expression of TLR4, TRIF, and TNF-α (Figures 5A,B). These results suggested that maternal HALEs diet may affect the expression of AMPK/mTOR/PPARα signaling pathway proteins, and lead to the disturbance of glycolipid metabolism in offspring.

### 3.4 Effects of maternal ALEs diet on gut microbiota in offspring

**Figure 6:**
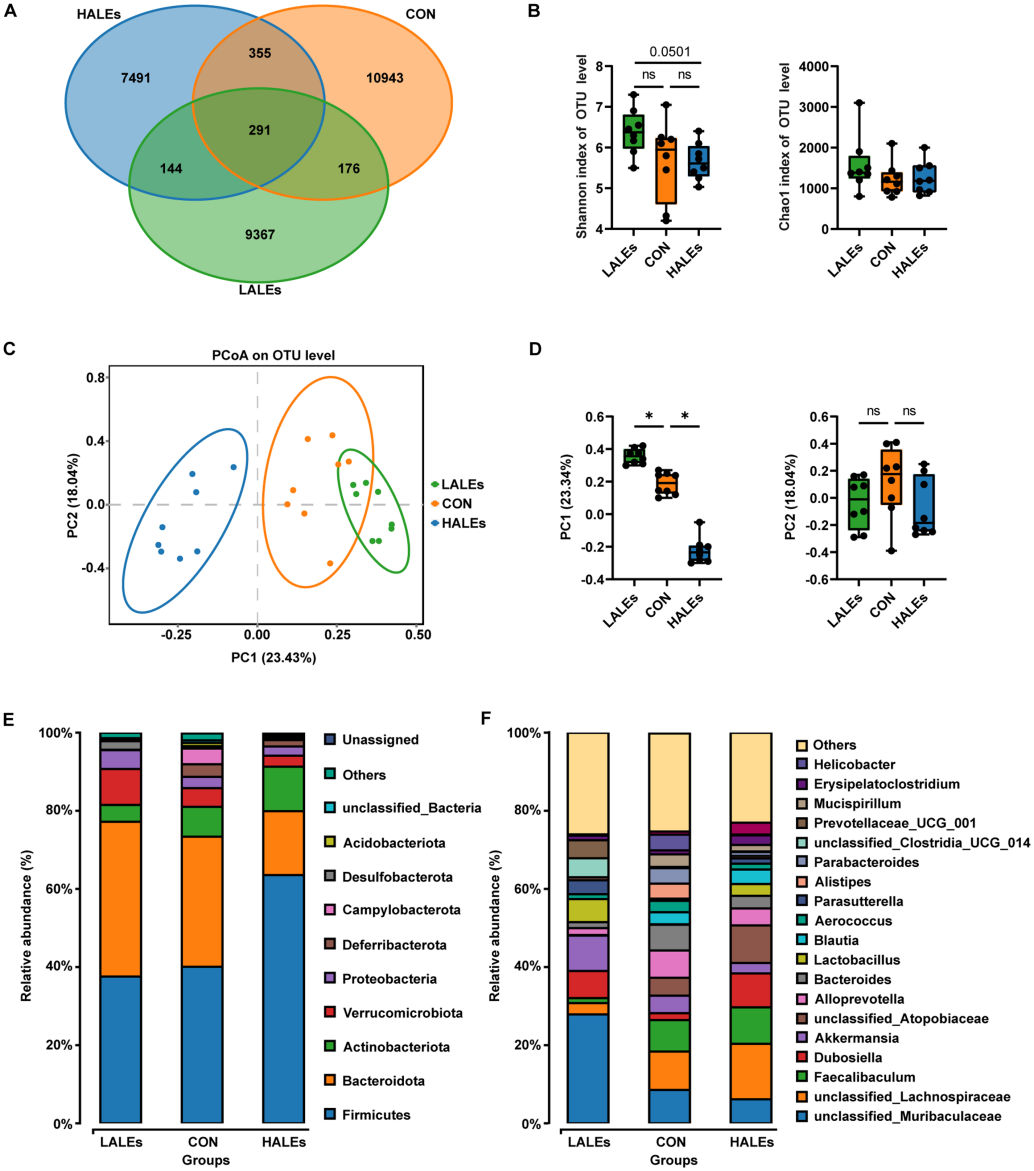
Maternal ALEs diet altered structures and composition of gut microbiota in offspring. **(A)** Venn diagram of the OTUs. **(B)** Shannon index of alpha diversity. **(C)** PCoA plots of gut communities. **(D)** PC1 and PC2 index of beta diversity. **(E)** Relative abundance of the bacterial population at the phylum level. **(F)** Relative abundance of the bacterial population at the genus level. *p < 0.05.

To investigate the impact of maternal ALEs diet on gut microbiota in offspring at weaning, we performed 16S rRNA sequencing of the cecal contents and the corresponding biological analysis. By analyzing the OTUs to assess shared and unique germs between the three groups, we found 291 shared OTUs, 10,943 unique OTUs in the CON group, 9,367 unique OTUs in the LALEs group, and 7,491 unique OTUs in the HALEs group (Figure 6A). The maternal HALEs diet had no significant effect on the alpha diversity of bacteria in the offspring (Figure 6B). The beta diversity measures, which characterizes differences between groups, showed significant separation of the gut microbiota between the three groups (Figures 6C,D).

**Figure 7:**
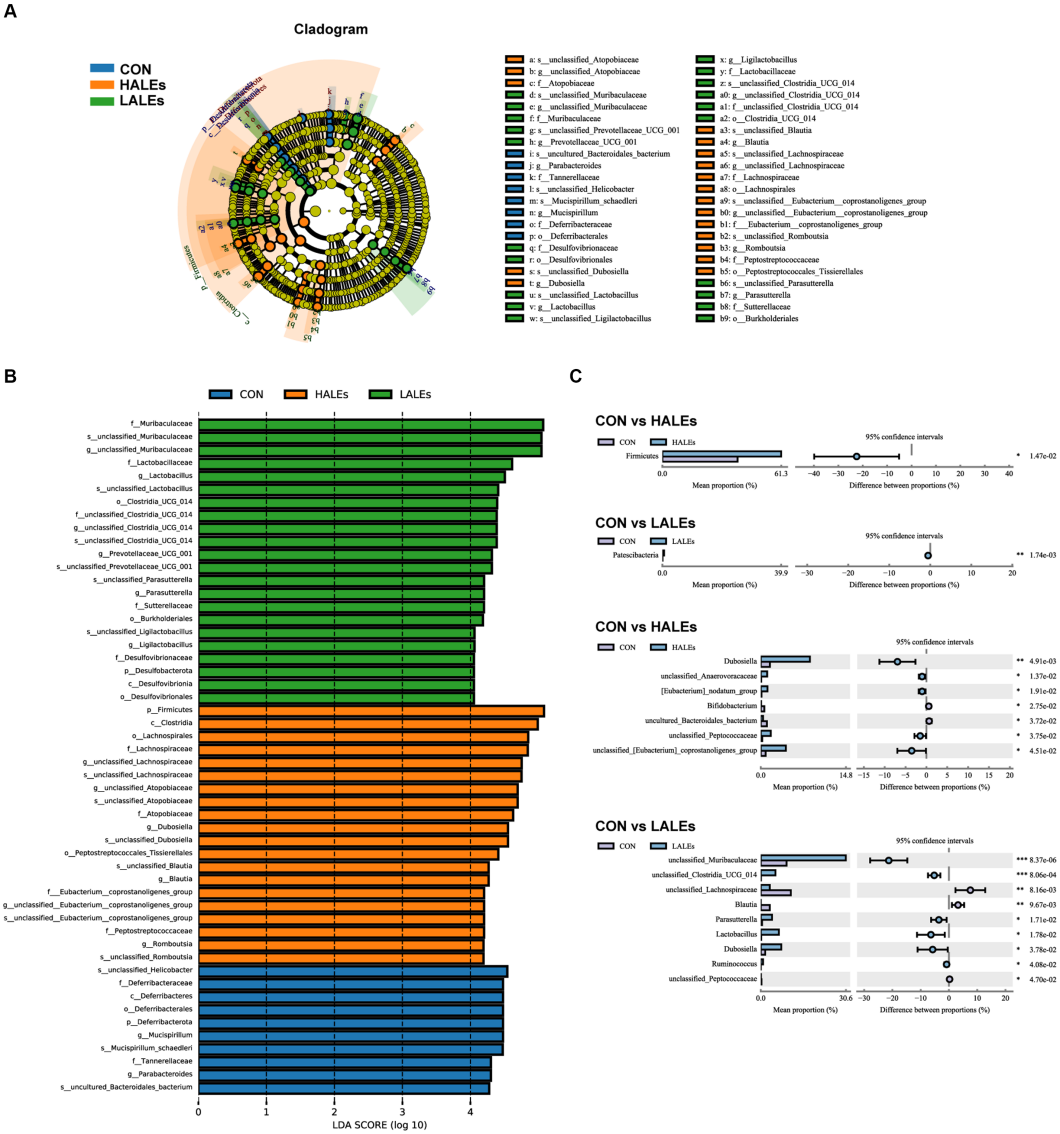
Key bacteria altered by the maternal ALEs diet in offspring **(A,B)**. LEfSe analysis of the gut microbiota from the phylum level to the genus level with LDA values of 4.0. **(C)** Wilcoxon rank-sum test bar plot on the phylum and genus level. *p < 0.05, **p < 0.01.

Then, we evaluated the relative abundance of gut microbial composition. The top 10 species at the phylum level were shown in Figure 6E, and the results have shown that Firmicutes was enriched in the HALEs diet offspring, followed by Bacteroidota, Actinobacteria, Verrucomicrobiota, and Proteobacteria, while Bacteroidota was enriched in the LALEs diet offspring, followed by Firmicutes, Verrucomicrobiota, and Proteobacteria. Besides, the top 20 species at the genus level with significant differences was shown in Figure 6F. To examine alteration in microbiota composition, the Wilcoxon rank-sum test was applied to analyze the differential species. Compared with the CON diet offspring, the maternal HALEs diet significantly increased the relative abundance of Firmicutes, and the maternal LALEs diet significantly increased the relative abundance of Patescibacteria at the phylum level. At the genus level, the maternal HALEs diet significantly increased the relative abundance of *Dubosiella,* unclassified_*Anaerovoracaceae*, *[Eubacterium]_nodatum*_group, unclassified_Peptococcaceae, unclassified *[Eubacterium]_coprostanoligenes*_grou*p*, and decreased the relative abundance of *Bifidobacterium*, uncultured_Bacteroidales_bacterium (Figure 7C).

**Figure 8:**
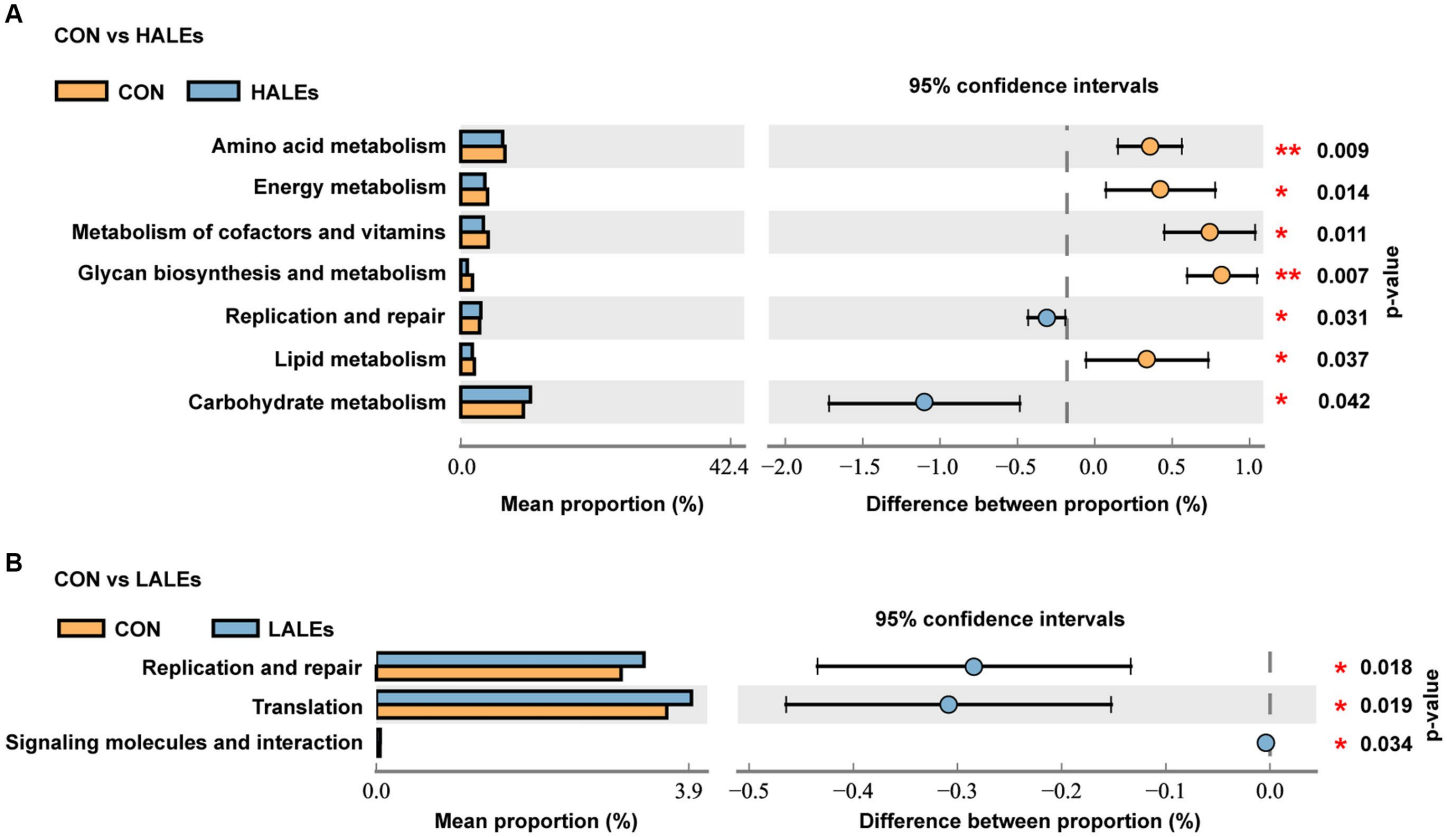
Metabolic pathways of bacterial communities by the KEGG pathway database in offspring. **(A)** Comparison of the HALEs and CON groups. **(B)** Comparison of the LALEs and CON groups. *p < 0.05, **p < 0.01.

To further assess the influence of each species on the differences between groups, we analyzed the gut microbiota using LEfSe (Figure 7A). The phylum Firmicutes, the class Clostridia, the order Lachnospirales, the family lachnospiraceae, and the genus unclassified_Atopobiaceae were the most abundant in the HALEs diet offspring. The species unclassified_*Helicobacter*, the family Deferribacteraceae, the class Deferribacteres, the order Deferribacterales, and the phylum Deferribacterota were the most abundant in the CON group (Figure 7B). In addition, the metabolic pathways of bacterial communities in offspring were analyzed by KEGG database (Figures 8A,B). Analysis of the composition and differences of metabolic pathways can reveal the changes of functional genes in the microbial community. On the level 2 of KEGG, replication and repair, and the carbohydrate metabolism pathway has been observed in HALEs diet offspring. Significantly, the low represented pathways in the HALEs group were mainly in biological processes associated with metabolism compared with the CON group. These pathways included amino acid metabolism, energy metabolism, metabolism of cofactors and vitamins, glycan biosynthesis and metabolism, and lipid metabolism (Figure 8A).

## 4 Discussion

With the increasing proportion of heat-processed foods in global food consumption, the onset age of metabolic diseases such as obesity and diabetes has gradually decreased (17). Chronic metabolic diseases that caused by disorders of glucose and lipid metabolism affect a vast number of individuals worldwide, and their occurrence is fetal (18, 19). Significantly, dietary ALEs are formed by modifying proteins with lipid oxidation products, and heat-processed foods contain a certain amount of ALEs. Our previous studies have shown that dietary ALEs induced liver damage by modulating hepatic lipid metabolism. Nevertheless, the transgenerational effects of dietary ALEs have been less studied. It has been found that the adverse outcomes caused by dietary intake of heat-processed foods are not limited to themselves, but may have a corresponding impact on the health of their offspring (20), revealing the transgenerational effects of harmful substances in heat-processed foods. Therefore, it is important to analyze the effects of dietary ALEs on offspring metabolism.

Dietary ALEs caused by non-enzymatic modification of proteins is a health risk factor in heat-processed foods (21, 22). Recent studies have shown that ALEs diet led to intestinal barrier breakdown, liver dysfunction and lipid accumulation, and ultimately liver injury (11). As the central organ of metabolism, liver not only participates in the synthesis of glycogen and fat, but also secretes regulatory factors that act on many major metabolic tissues to synergistically regulate glycolipid metabolism (23). In this study, the levels of blood glucose and serum TC/TG/LDL-C in dams of HALEs group were significantly higher than in those of CON group. We found that ALEs-rich diet caused glucose and lipid metabolism disturbance and even resistance in dams, which is consistent with previous research. In addition, it is worth noting that the offspring of HALEs group at weaning displayed increased body weight and glucose intolerance. These results suggested that the maternal ALEs diet could cause glucose metabolism disorders, which would help create an adverse developmental environment for offspring *in utero* and early postpartum period.

In terms of the metabolism, we found that maternal ALEs-rich diet increased body weight, blood glucose, and insulin levels of the offspring, and the offspring exhibited glucose intolerance. The insulin concentration was elevated, not at the level of insulin resistance, but it is nonetheless of concern. In addition, higher levels of serum TC, TG, LDL-C, and leptin were observed in the offspring of HALEs group, indicating that maternal ALEs-rich diet induced hyperlipidemia. Leptin is a hormone secreted by fat tissue, and its content in the serum is proportional to the size of the fat tissue. It plays a key role in glucose homeostasis, maintenance of fat tissue, and immune function (24). Leptin resistance and obesity-associated hyperleptinemia are associated with insulin resistance, type 2 diabetes, and diabetic vascular complications (25). Insulin and leptin were reliable biomarker of glycolipid metabolism disorders (26). Furthermore, glycogen is the main intracellular storage form of glucose in the liver, and glycogen levels are thought to be associated with insulin resistance in mice (27). The AB-PAS staining in the liver further confirmed the effect of HALEs diet on the hepatic glycogen content of offspring. The insulin and leptin levels of HALEs diet offspring increased significantly, suggesting that maternal ALEs diet adversely affects the glucose and lipid homeostasis of offspring.

Pathological staining of the liver showed that maternal ALEs diet could increase hepatic glycogen and lipid content in offspring, which indicated dietary ALEs might regulate the liver glycolipid metabolism of offspring. To further investigate the molecular mechanism of maternal ALEs diet, the changes of protein expression in the liver tissue of offspring were detected. The results showed that maternal ALEs-rich diet disrupted the glycolipid metabolism and reduced insulin sensitivity of offspring mice via AMPK/mTOR/PPARα pathways. As expected, the glycolipid metabolism of HALEs diet offspring was significantly impaired. Recent studies have demonstrated that the offspring of suboptimal maternal nutrition were obese at weaning, and accompanied by decreased gene expression for PPARα (28). Consistently, we found that the PPARα was significantly decreased in the offspring of HALEs group. Therefore, the liver PPARα pathway is an important signaling pathway for maternal diet to regulate offspring’s glycolipid metabolism (29). As a core transcriptional regulator of glycolipid metabolism (30), PPARα could inhibit the release of inflammatory factors, resist oxidative stress, improve insulin resistance and regulate lipid metabolism by inducing transcription of downstream target genes (mainly PKM2/IRS-1) (31, 32). Studies have found that PKM2 is a key regulatory enzyme in glucose metabolism, and IRS-1 mediates the metabolic and growth-promoting functions of insulin, both of which are major factor contributing to impaired glucose transport (33). In addition, activation of TLR4/TRIF signaling pathway has been found to be closely associated with hepatic lipid accumulation (34) and elevated concentrations of TNF-α in liver tissue are thought to be associated with liver inflammation, glucose and insulin disturbances (35). In the present study, maternal ALEs-rich diet induced AMPK activation, followed by mTOR and PPARα inhibition in offspring mice. Our results showed that maternal ALEs diet regulated the glycolipid metabolism of offspring via AMPK/mTOR/PPARα pathways.

A growing body of evidence suggests that the offspring microbiome disorders influenced by maternal diet plays an important role in glycolipid homeostasis (36). Given that the gut microbiota of offspring can be altered by maternal diet (37), we investigated the effects of maternal ALEs diet on the structure and diversity of the gut microbiota in offspring. The study found that maternal HALEs diet had a negative effect on glucose and lipid metabolism in offspring mice and the gut microbiota at weaning. The principal co-ordinates analysis (PCoA) showed that both the HALEs and LALEs groups were significantly different from the CON group. The results indicated that the maternal ALEs diet had a certain effect on the beta diversity of bacteria in the offspring. From the relative abundance of gut microbial composition, it can be observed that maternal HALEs diet resulted in significant changes in the gut microbiota of offspring, characterized by an increased Firmicutes/Bacteroidetes ratio at the phylum level. Previous studies have shown that the relative proportion of Bacteroidetes was reduced in obese mice (38), and the changes in Firmicutes/Bacteroidetes ratios have been closely associated with metabolic disorders, such as obesity and diabetes (39). In this study, the maternal HALEs diet significantly increased the abundance of *Dubosiella,* and *Dubosiella* had significantly increased abundance in high fat diet feeding mice, suggesting its specific role in the obesity-related metabolic phenotype (40). In addition, the relative abundance of *Bifidobacterium* and uncultured_Bacteroidales_bacterium was significantly decreased in the HALEs group. Claims have been made for positive effects of *Bifidobacterium* on infant growth and health (41). *Bifidobacterium* was important in obesity and type 2 diabetes, and may be involved in the dietary carbohydrate - microbiome - host metabolic axis (42). Bacteroidales has been reported to be a probiotic bacterium, exercising a beneficial effect on the gut microbiota (43). Bacteroidales could stimulate the production of fucosylated glycans in the gut (44). In addition, The bacteria also stimulates angiogenesis in the newborn epithelium (45), which enhances the body’s absorption of nutrients. Therefore, the altered gut microbiota of HALEs diet offspring may have a role in the promotion of susceptibility to obesity and diabetes when they encounter metabolic stress in later life. The relationship between intestinal microecological disorders and metabolic disorders may be related to metabolites derived from gut microbiota, which is worthy of further study.

In conclusion, our results showed that maternal ALEs diet negatively affects not only the metabolic homeostasis of dams, but also the glycolipid metabolism and gut microbiota of offspring. The maternal ALEs-rich diet may induce hepatic glycolipid accumulation, abnormal liver function, and disturbance of metabolism parameters in offspring through the AMPK/mTOR/PPARα signaling pathway. However, the causal relationship between maternal ALEs diet, regulation of gut microbiota, and metabolic parameters in offspring remains unclear, and more experiments on fecal microbiota transplantation and antibiotic intervention are needed to investigate the exact association.

## Data availability statement

The data presented in the study are deposited in the NCBI SRA repository, accession number PRJNA1119259.

## Ethics statement

The animal study was approved by international ethical guidelines and the Institutional Animal Care and Use Committee of Nankai University. The study was conducted in accordance with the local legislation and institutional requirements.

## Author contributions

WP: Conceptualization, Data curation, Investigation, Writing – original draft. BZ: Formal analysis, Investigation, Writing – original draft. JZ: Methodology, Project administration, Writing – review & editing. TC: Methodology, Visualization, Writing – review & editing. QH: Data curation, Visualization, Writing – review & editing. ZY: Funding acquisition, Supervision, Writing – review & editing.
